# Assessing the Ecological Conversion Efficiency of Chub Mackerel, *Somber japonicus*, in Wild Conditions Based on an In Situ Enriched Simulation Method

**DOI:** 10.3390/ani13203159

**Published:** 2023-10-10

**Authors:** Xin Sun, Miao Yu, Qisheng Tang, Yao Sun

**Affiliations:** Key Laboratory for Sustainable Utilization of Marine Fisheries Resources of Ministry of Agriculture, Yellow Sea Fisheries Research Institute, CAFS, 106 Nanjing Road, Qingdao 266071, China; sunxin@stu.ouc.edu.cn (X.S.);

**Keywords:** ecological conversion efficiency, gastric evacuation rate, in situ simulation method, chub mackerel

## Abstract

**Simple Summary:**

Chub mackerel is an economic fishery species that is key in transferring energy from zooplankton to large-bodied carnivores through the local food webs. Understanding the ecological conversion efficiency of this species is important for ecosystem-based fishery. However, the ecological conversion efficiency cannot be accurately determined in the laboratory. In this study, an in situ enrichment method was designed to simulate the wild conditions and used for the determination of ecological conversion efficiency of chub mackerel. Additionally, the method described in this study provided insights into the determination of other wild fishes that are difficult to sample in situ and domesticate in the laboratory.

**Abstract:**

Understanding the ecological conversion efficiency of a fish species can be used to estimate the potential impact of the marine food web and accordingly provides scientific advice to ecosystem-based fishery management. However, only laboratory experiments may limit the accuracy of determining this index. In this study, food ingestion and ecological conversion efficiency of wild chub mackerel (*Somber japonicus*), a typical marine pelagic fish, were determined with gastric evacuation method in laboratory and in situ enriched simulation conditions. Additionally, the effect of temperature and body weight on ecological conversion efficiency was further estimated based on the 2D interpolation method. The results showed that, at 25.1 °C, the ecological conversion efficiency determined in-lab (35.31%) was significantly higher than in situ (23.85%). Moreover, the interpolation model estimated that with an increase in temperature (10–27 °C), the ecological conversion efficiency initially decreased, followed by an increase when the temperature reached 18 °C, but the ecological conversion efficiency generally decreased against the body weight at each temperature. The findings of this study enhanced the understanding of the energy budget of chub mackerel and also provided an efficient method for the determination of wild fishes that are difficult to sample in situ and domesticate in the laboratory.

## 1. Introduction

The trophodynamics focuses on the analysis of quantitative relations of energy flow and transfer among prey and predators in food webs [1,2]. Ecology and fisheries research has documented and sought to explain patterns of trophodynamics through food webs over decades [3,4,5,6], which is also one of the important subjects of the Global Ocean Ecosystem Dynamics (GLOBEC). The productive capacity of upper trophic levels can be constrained by the efficiency of energy transfer from the lower trophic levels, which in turn limits the biomass of the upper levels and the number of trophic transfers between basal resources and top predators [7,8].

The quantification of ecological efficiency depends on the ecological conversion efficiencies, e.g., the ratio of energy allocated to growth from food ingestion for one species in a certain trophic level [9,10]. Unlike traditional fishery advice which commonly derives from population-based indices, such as abundance, age structure, and, condition index, the ecological conversion efficiency describes the energetic budget of a species and its potential transfer rate through the food web. The understanding of the ecological conversion efficiencies of a species can be used to estimate the potential impact of population variations on other species in an adjacent trophic level and the food web through a cascading effect [11,12], thereby providing scientific advice to the ecosystem-based fishery management. However, the accurate determination of the ecological conversion efficiency of a certain fish species could be difficult because this index is a dynamic variable that depends on the co-effect of both fish themselves and the environmental factors. Nevertheless, previous studies have investigated that body weight and temperature are the most essential factors of the ecological conversion efficiency of a fish species [9,10]. On one hand, fish body weight is closely related to the different metabolic rates per unit weight at different developmental stages of the fish [13]. On the other hand, temperature determines the physiological and biochemical functions, such as metabolic rate, digestion, enzymatic activity, and gastric motility. These variables could be significantly different under different temperatures [14].

Chub mackerel, *Somber japonicus*, is a typical marine pelagic fish widely distributed in the North Pacific and North Atlantic Oceans [15]. In China, this species plays an irreplaceable role in Chinese food culture and also has been previously reported as a keystone species in the Yellow Sea food web [9]. Chub mackerel prefers zooplankton and mesopelagic fauna such as *copepoda* and *euphausiidea* as prey [16]. Predators of chub mackerel include large-bodied carnivores and marine mammals [17,18]. Accordingly, this species functions as a connector that links the energy from low trophic to high trophic levels through the local food web. This emphasizes the importance of investigation of the ecological conversion efficiency of chub mackerel.

Laboratory experiments are widely used in the determination of the indices for a variety of fishes due to their excellent characteristics such as easy-to-control experiment conditions, low experiment fare, and relatively simple operation procedures [19]. Nevertheless, due to limited by the monotonous laboratory conditions, it is difficult to fully simulate the natural environment for aquatic wildlife. Therefore, the ecological conversion efficiency of animals in wild environment cannot be accurately determined from laboratory-based experiments. Similar to most natural growth species that feature multiple wild habits, chub mackerel is difficult to sample in situ and domesticate in the laboratory [20]. This limits the determination of the biological processes of this species and accordingly weakens the understanding of energy flow in the food web.

In this study, an in situ experiment was designed by using a holding net cage that can be submerged into the seawater for holding the wildly caught chub mackerel. Ecological conversion efficiency was calculated based on the fish indices including gastric evacuation rate, daily growth rate, and daily ingestion rate that were determined in the laboratory and in in situ, respectively. Additionally, the effect of temperature and body weight on the ecological conversion rate was further estimated. The aims of the present study are as follows: (1) determine the growth, ingestion, and ecological conversion efficiency of chub mackerel in the wild environment; (2) find out an easy-to-operate method that enables the determination of indices in wild fishes; and (3) identify the potential uncertainty of using the laboratory experiment to represent the natural condition. The studies on the in situ determination of ecological conversion efficiency for wild fishes are rare. The finding of the present study can fill the knowledge gap of energy flow of chub mackerel through the marine food web, providing advice for food web-based fisheries management, and the methods of the present study could provide insights into the determination of wild fishes.

## 2. Materials and Methods

### 2.1. Fish Collection and Acclimation

**Figure 1:**
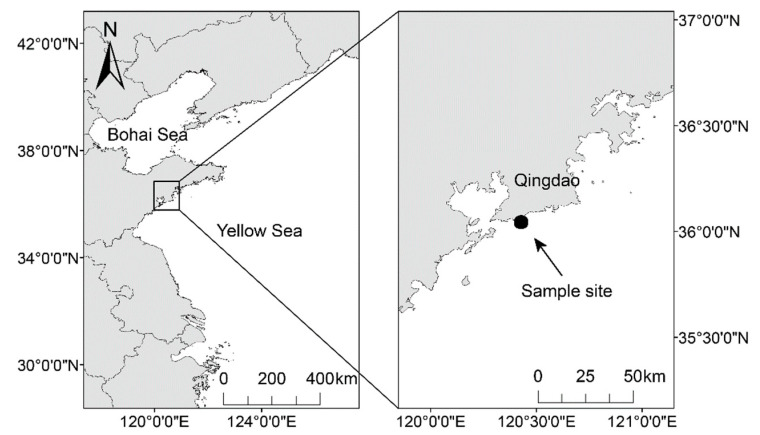
Location of the site where the wild chub mackerel were collected and the in situ simulation experiment.

Chub mackerel were collected from their main oviposition migration area on the southern coast of the Shandong peninsula, China (Figure 1), in May 2002, when many young fish are widely distributed to obtain food. For collection, a 6 × 6 × 4.5 m catching net was submerged into the water about 2 m from where fish were gathered, with lures placed in the middle. When several fish moved in, the edge of the net was drawn. This method could minimize the injury caused to fish during catching. In this study, approximately 1200 individuals were collected.

A holding cage (12 m × 6 m × 4.5 m with mesh size of 10 mm) that could be submerged in the seawater was designed for holding the caught chub mackerel in situ. Approximately 400 of the collected fish were randomly collected and then gently removed from the catching net into the holding cage, and the rest of the fish were removed into 10 laboratory tanks (2.5 m × 1 m × 1 m), each tank including approximately 80 individuals. In this case, the fish that were kept in holding cage in natural seawater were recorded as “in situ”, while those kept in the laboratory tanks were recorded as “in-lab”, respectively. Water in the laboratory tanks was collected from the same place where fish were collected and kept at the same temperature (25.1 ± 0.8 °C). All the fish were acclimated for another 7 days. During acclimation, fish were fed twice daily at 7:30 and 16:30 with surimi of a species of marine small-sized fish, *Pseudosciaena polyactis*. Fish were overfed (with residue food occurrence) each time to ensure maximum feeding level.

### 2.2. Experiment Procedure

In order to compare the indices of chub mackerel in-lab and in situ, the same procedures were applied for the fish in each condition. Gastric evacuation rate represents the rate at which food is digested in the stomach after ingestion [21]. For determination, every 10 individuals were randomly collected from the holding tank every 1 h, and this was performed 11 times. Once collected, fish were immediately euthanized with 10% formalin, then measured to determine folk length, whole weight, and excised stomach tissue (formalin was not prohibited for euthanasia of fish as per the legislature of the region in the year of experiment). Stomach tissue, stomach content, and fish food samples was dried at 70 °C in an oven for 48 h before weighing on a pre-weight culture dish, then smashed and sieved, and then analyzed for the total carbon and energy content using the element analyzer (P-E240C) and calorimeter (XYR-1), respectively.

Daily growth rate and daily ingestion rate for both in situ and in-lab fish were determined. In this experiment, continuous 24 h determination was conducted every 6 days, and this was performed five times (30 days in total). On each sample day, five individuals were randomly sampled at 0:00, 3:00, 6:00, 9:00, 12:00, 15:00, 18:00, 21:00, and 24:00. Sampled individuals were treated with 10% formalin at once and then their body weight and food content in the stomach were determined.

Another experiment was designed to estimate the ecological conservation rate under different temperature and body weight. Another three temperature levels, 26, 18, and 10 °C were applied, as they represented the average temperature in summer, autumn, and winter of the sampling area. This experiment was only applied to in-lab fish because it is impossible to control the nature seawater temperature. In this experiment, the collected fish (ranging from 30–170 g) were divided into eight groups (30, 50, 70, 90, 110, 130, 150, 170 g) based on their body weight. Those groups containing less than 10 individuals were excluded from the analysis as insufficient sample size for determination of ecological conversion efficiency. Fish were removed into an experiment tank (same size and conditions as the holding tank). For acclimation, external thermostatic devices were placed on the outer side of the tanks, to control the water temperature reduction at a rate of approximately 0.2 °C per hour. The fish were kept for another 7 days after the target temperatures were reached. The same procedures were used for the determination of ecological conversion efficiency.

### 2.3. The Calculation of Fish Indices

Gastric evacuation rate is indicated by the slope of the regression equation between the stomach content over time. In this study, natural logarithm was used as the regression model because this model could best fit with the gastric evacuation of the species [10]. The instantaneous gastric evacuation rate (R_t_) was obtained from the slope of the linear regression equation between natural logarithm values of instantaneous food contents in the stomach and their corresponding time. In order to avoid the weight difference among each individual, the %stomach content was calculated by dividing the stomach content weight by the body weight.

Daily ingestion rate was calculated according to the Eggers model [22]:

C_d_ = 24 × S × R_t_ (1)

where C_d_ represents the daily ingestion rate, and S represents the average food content in the stomach during the continuous 24 h determination.

Equation (2) was used to calculate ecological conversion efficiency (E_g_) [9]:

E_g_ = (C_d_/G_d_) × 100% (2)

where G_d_ is the daily growth rate that is obtained from the linear regression equation describing the change in the fish weight in different experiment periods.

### 2.4. Data Analysis

A matrix-based 2D interpolation method was used to extend the body weight and temperature covering various levels within the selected ranges. In this method, another two temperatures (14 and 22 °C) were inserted into the original three temperature levels (10, 18, and 26 °C). In this case, a 5 × 8 matrix was built with the five temperature levels representing the first axis and the eight body weight levels representing the second axis. Then, 17 determined ecological conversion efficiency were first filled into the 5 × 8 matrix, and the rest 23 values were estimated with 2D interpolation using the method “Spline” in OriginPro 2021 (Table 1). The interpolation method could minimize the number of sacrificed fish in the experiment.

The ecological conversion efficiency at 25 °C estimated from the interpolation method was extracted, and then compared with the determined ecological conversion efficiencies from both in-lab and in situ samples. The comparison between estimated and in-lab determined ecological conversion efficiency was used to verify the performance of the simulation, while the comparison between estimated and in situ determined ecological conversion efficiency was used to identify the potential error of using the laboratory experiment to represent the natural condition.

## 3. Results

### 3.1. Gastric Evacuation Rate

**Figure 2:**
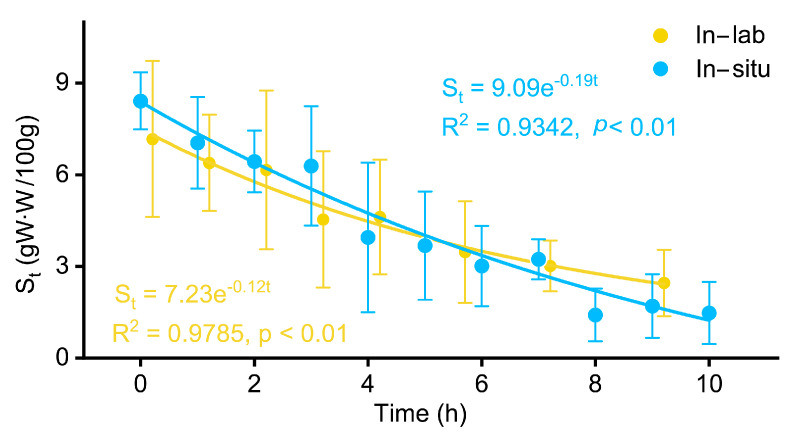
The instantaneous gastric contents (S_t_) during the experiment period and their fitting curves of chub mackerel kept in-lab and in situ, respectively.

Clear differences in the decrease rate of instantaneous food content in the stomach (S_t_) were observed between the chub mackerel in-lab and in situ (Figure 2). The slope of natural logarithm regression between the S_t_ and time (t) for chub mackerel in-lab (slope = 0.12) was overall lower than that in situ (slope = 0.19). The regression equations for in-lab and in situ are described as follows:

S_t_ (in-lab) = 7.23e^−0.12t^

S_t_ (in situ) = 9.09e^−0.19t^

### 3.2. The Determined Fish Indices

**Figure 3:**
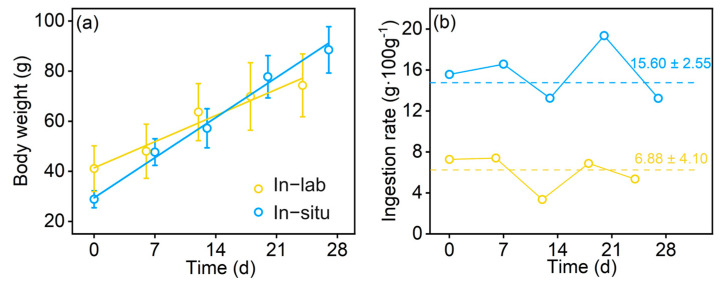
Growth (**a**) and ingestion rate (**b**) of chub mackerel kept in-lab (yellow) and in situ (blue).

The body weight of in-lab fish increased at an obviously lower rate than in situ fish during the experiment period (Figure 3a). In-lab fish grew from 41.06 ± 9.132 g to 74.33 ± 12.54 g, while in situ fish grew from 28.85 ± 3.39 g to 88.51 ± 9.26 g. Additionally, the daily ingestion rate (C_d_) calculated from the Eggers model showed the similar fluctuation between the fish that were kept in-lab and in situ (Figure 3b). The average daily ingestion rate of in-lab fish (6.88 ± 4.10) was significantly lower than that of the in situ fish (15.60 ± 2.55) (ANOVA *p* < 0.01). The ecological conversion efficiency of chub mackerel in-lab and in situ are 35.31% and 23.85%, respectively.

### 3.3. The Effect of Temperature and Body Weight on the Ecological Conversion Efficiency

**Figure 4:**
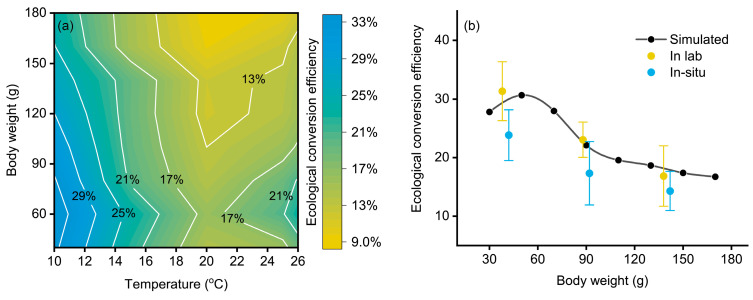
The simulated ecological conversion efficiency under the influence of temperature and body weight (**a**), and the comparison between the simulated (black), in-lab (yellow), and in situ (blue) ecological conversion efficiencies at 25 °C (**b**).

The daily growth rate and the daily ingestion rate at 10 °C were significantly lower than that in 26 and 18 °C (ANOVA *p* < 0.01) (Table 2). However, the ecological conversion efficiency was lowest at 18 °C while highest at 10 °C. The overall ecological conversion efficiency estimated from temperature (10–26 °C) and body weight (30–60 g) matries ranges from 9 to 33% (Figure 4a). In general, the ecological conversion efficiency decreases against the body weights at each temperature, except for those body weight from 30~50 g that showed an increase in body weight. For the chub mackerel with various body weights, the lowest ecological conversion efficiency (approximately 9–17%) was observed at approximately 18–24 °C, while ecological conversion efficiency reached a relatively high level when the temperature was below 15 °C and above 26 °C.

As both in-lab and in situ experiments were performed under 25 °C and shared the body weight of fish of around 40, 90, and 130 g, the determined ecological conversion efficiency of fish was used and compared with the estimated ecological conversion efficiency that was extracted at the same temperature and at the same body weight. The ecological conversion efficiency of the in-lab fish was close to that of the simulation, and the simulated values were within the error bars of the determination at each selected body weight (Figure 4b). However, the ecological conversion efficiency of the in situ fish was overall lower than the simulated value, although the ecological conversion efficiency also observed a decrease with body weight.

## 4. Discussion

This study determined the daily growth rate and daily ingestion rate of chub mackerel, *S. japonicus*, based on gastric evacuation under in-lab and in situ conditions, and estimated the ecological conversion efficiency under the effect of temperature and body weight using the 2D interpolation matrix method. Through the comparison of the indices, this study demonstrated the effects of the in-lab and in situ conditions on the energetic process of chub mackerel, providing insights into the determination of the energy dynamics process of wild fishes.

### 4.1. The Differences in the Indices between In-Lab and In Situ Conditions

In this study, large differences in gastric evacuation rate, daily growth rate, and daily ingestion rate were observed between the in-lab and the in situ fish, which confirmed the importance of in situ experiments in the determination of the fish indices. The gastric evacuation rate for fish is commonly considered to be largely affected by temperature and body weight, which are closely related to the different metabolic rates [21,23,24]. Although the in-lab and in situ fish in the present study were similar in terms of both temperature and body weight, they still showed differences in gastric evacuation rate, growth rate, and ingestion rate. The differences in these indices could be attributed to the holding environment. It has been previously reported that gastric evacuations can coincide with the recovery of appetite for most fish [13,25]. In other words, the higher gastric evacuation rate should correspond to the higher ingestion rate. Additionally, feeding fish at lower gastric fullness can significantly increase their growth [26]. This supports the results of the present study where in situ fish represented both high gastric evacuation rate, daily ingestion rate, and daily growth rate compared to in-lab fish. Moreover, chub mackerel exhibits intense characteristics in terms of activities including associating groups and hunting for food in swimming state [27]. Such activities were obvious for those fish that were held in the in situ cage but not significant when they were held in the laboratory tanks. Therefore, it is inferred that the differences in indices of fish that were held in-lab and in situ may result from their energy spent on activities and accordingly facilitating the accuracy of determined ecological conversion efficiency of this species in natural environment.

It is understood that a laboratory experiment may not fully represent the natural field conditions due to limited sources of variation. This is also observed in the present study where the overall in-lab ecological conversion efficiencies demonstrated overestimation. The in situ experiment is closer to the natural condition and enables a more accurate determination of the energetic processes of the wild fishes, and it still cannot fully mimic the fish’s natural habits. Nonetheless, it still offers insights into the overall trend as determined by the result of in situ fish, which confirms the representativeness of the laboratory experiment.

### 4.2. The Ecological Conversion Efficiency of Chub Mackerel

The co-effect of temperature and body weight on ecological conversion efficiency in this study was based on interpolation. It is important to note that this method may lose accuracy in extending the observation results to matrix or contour map, particularly when using less determined data to interpolate a larger matrix. However, in this study, the data determined in each body weight group covered most of the temperature axis, and only two temperatures were inserted into the original three temperature levels, which could reduce the biases in building the matrix.

The ecological conversion efficiency of most fish ranges from 10–30% [9]. Although generally within this range, the ecological conversion efficiencies of chub mackerel were significantly impacted by temperature and body weight. The chub mackerel show strong swimming habits, which consume large proportions of the energy budget. The swimming sphere of the in-lab chub mackerel was limited by the relatively small tanks, leading to a higher energy that can be reserved in their body and accordingly cause overall higher ecological conversion efficiencies than the in situ fish.

Body weight exhibited a negative correlation with ecological conversion efficiency in chub mackerel. As body weight increased, the required energy for maintaining body structure, metabolism, and activity also increased [28,29]. Additionally, the specific growth cost is also higher for heavier fish [30]. Therefore, when specific ingestion is constant, lighter individuals could allocate more energy to growth, resulting in higher ecological conversion efficiency compared to heavier individuals.

At low temperatures, both growth and ingestion rates decreased significantly, while ecological conversion efficiency largely increased. This is because reducing the metabolic rate and activities are commonly adopted by many eurythermal fishes when facing low temperatures [31]. This strategy allows allocation of more energy into body reserve [32], therefore achieving higher ecological conversion efficiencies. On the other hand, around 20 °C is considered as the optimal temperature with the highest metabolic rate for chub mackerel [33] but represented the lowest ecological conversion efficiency that was determined in this study. The similar pattern is also confirmed by previous studies on Atlantic cod (*Gadus morhua*) [34], European sprat (*Sprattus sprattus*) [35], sea bass (*Acanthistius patachonicus*) [36], and steelhead trout (*Oncorhynchus mykiss*) [13]. For most fishes, the optimal temperature commonly represents the highest proportion of energy costs for activity and metabolism [37]. Due to this, the increased food ingestion can be regarded as the guarantee of the requirement of the increased energy costs. However, it is worth noticing that all the fish were overfed during the experiment. Fish in wild environments cannot always remain in this satiety state. The increased metabolism and activity at optimal temperature is considered as a strategy for fish to obtain more food [38]. Therefore, we infer that under the wild environment, the reduced pattern of ecological conversion efficiency at optimal temperature may be mitigated.

The ecological conversion efficiencies of the in-lab fish were overall higher than those of the in situ fish. As mentioned above, this mainly results from the differences in the portion of energy spent on activities. However, the differences between the two methods decrease when the body weight increases. This may be due to the fact that heavier individuals are also more robust, which means they could spend less proportion of energy on achieving the same behaviors. As a result, we assume that the uncertainties that using in-lab experiments to represent wild conditions could be mitigated to a certain extent with an increase in body weight. It should be noted that in addition to body weight and temperature, ecological conversion efficiency could be also influenced by various other factors associated with the complex natural environment, such as food diversity, aggregation behavior, and migration.

The ecological conversion efficiency indicates the trophodynamics of the bottom-to-top food web in an ecosystem. Overestimation of ecological conversion efficiency of one trophic level may mislead the total allowable catch system (TAC), exceeding the ecosystem’s limitation, thereby negatively impacting the marine food web. Chub mackerel has been receiving increasing attention as a new target fishery species in the Yellow Sea, and the reasonable procurement of this species is accordingly the key for ensuring the sustainability of the regional ecosystem. The present study indicates that although the laboratory experiment could reflect the trends of the ecological conversion efficiency in response to the changing temperature and body weight, it may overestimate the ecological conversion efficiency of chub mackerel in the wild environment. This is worth considering in the food web-based fishery management.

## 5. Conclusions

In situ chub mackerel illustrated better appetite and higher growth rate compared to the in-lab ones. Chub mackerel in the in situ enriched condition featured lower ecological conversion efficiency, as this condition allows their active behaviors, which enable more energy that can be spent on activities. As a result, this value is considered closer to the indices of the fish in the natural environment than that calculated for the laboratory-based condition. The ecological conversion efficiency generally decreases against the body weight, suggesting that the uncertainties of using in-lab experiments to represent wild conditions could be mitigated to a certain extent with an increase in body weight. Nonetheless, the overall in-lab determined ecological conversion efficiencies demonstrated overestimation, which should be considered in the food web-based fishery management.

## Figures and Tables

**Table 1:** The matrix contains determined (bold) and estimated ecological conversion efficiencies across body weights and temperature levels.

		Body Weight							
		30 g	50 g	70 g	90 g	110 g	130 g	150 g	170 g
Temperature	10 °C	** 33.70 **	** 35.01 **	** 33.56 **	** 31.29 **	29.06	26.93	** 25.15 **	24.02
	14 °C	20.52	22.06	20.25	19.33	18.01	17.40	14.98	14.11
	18 °C	** 14.2 **	** 16.03 **	** 14.11 **	12.97	** 12.31 **	** 12.19 **	** 9.72 **	** 8.97 **
	22 °C	19.48	21.50	19.12	15.92	14.29	14.14	12.61	11.69
	26 °C	31.8	** 34.22 **	** 31.76 **	** 24.33 **	21.50	** 20.23 **	20.44	** 19.21 **

**Table 2:** Daily growth rate (G_d_), daily ingestion rate (C_d_), and ecological conversion efficiency (Eg) under 26, 18, and 10 °C. Significant differences are tested with ANOVA and marked as different letters.

	26 °C	18 °C	10 °C
G_d_	22.36 ± 3.23 ^a^	18.80 ± 4.98 ^a^	7.34 ± 0.94 ^c^
C_d_	110.54 ± 6.23 ^a^	119.78 ± 7.60 ^a^	22.39 ± 1.92 ^c^
E_g_	20.23 ± 2.03% ^a^	15.86 ± 5.17% ^b^	33.07 ± 7.01% ^c^

## Data Availability

Available from the corresponding author upon reasonable request.

## References

[1] Ríos M.F., Venerus L.A., Karachle P.K., Reid W.D., Erzini K., Stergiou K.I., Galván D.E. (2019). Linking size-based trophodynamics and morphological traits in marine fishes. Fish Fish..

[2] Bas M., Briz I.G.I., Alvarez M., Vales D.G., Crespo E.A., Cardona L. (2019). Back to the future? Late Holocene marine food web structure in a warm climatic phase as a predictor of trophodynamics in a warmer South-Western Atlantic Ocean. Glob. Chang. Biol..

[3] Tang Q., Su J., Kishi M.J., Oh I.S. (2007). An introduction to the Second China–Japan–Korea Joint GLOBEC Symposium on the ecosystem structure, food web trophodynamics and physical–biological processes in the Northwest Pacific. J. Mar. Syst..

[4] Lawton J., May R. (1984). Fisheries Genetics, dynamics and politics. Nature.

[5] Berdnikov S., Selyutin V., Vasilchenko V., Caddy J. (1999). Trophodynamic model of the Black and Azov Sea pelagic ecosystem. Fish. Res..

[6] Stratton M.A., Nesslage G.M., Latour R.J. (2019). Multi-decadal climate and fishing predictors of abundance for U.S. South Atlantic coastal fishes and invertebrates. Fish. Oceanogr..

[7] Natugonza V., Ogutu-Ohwayo R., Musinguzi L., Kashindye B., Jónsson S., Valtysson H.T. (2016). Exploring the structural and functional properties of the Lake Victoria food web, and the role of fisheries, using a mass balance model. Ecol. Model..

[8] Jayasinghe R.P.P.K., Amarasinghe U.S., Newton A. (2017). Evaluation of status of commercial fish stocks in European marine subareas using mean trophic levels of fish landings and spawning stock biomass. Ocean Coast. Manag..

[9] Tang Q., Guo X., Sun Y., Zhang B. (2007). Ecological conversion efficiency and its influencers in twelve species of fish in the Yellow Sea Ecosystem. J. Mar. Syst..

[10] Sun Y., Liu Y., Liu X., Tang Q. (2010). The influence of particle size of dietary prey on food consumption and ecological conversion efficiency of young-of-the-year sand lance, *Ammodytes personatus*. Deep-Sea Res. Part II.

[11] Carlucci R., Capezzuto F., Cipriano G., D’Onghia G., Fanizza C., Libralato S., Maglietta R., Maiorano P., Sion L., Tursi A. (2020). Assessment of cetacean–fishery interactions in the marine food web of the Gulf of Taranto (Northern Ionian Sea, Central Mediterranean Sea). Rev. Fish Biol. Fisher..

[12] Félix L., Vieira R., Monteiro S.M., Venâncio C. (2023). A meta-analytic review of monoterpene for fish anaesthesia. Fish Fish..

[13] Liu R., Zhou Y., Li Z., Huang M., Li L., Gao Q., Dong Y., Dong S. (2022). Evaluation of the effects of temperature on gastric evacuation and the associated mathematical models in different sizes steelhead trout (*Oncorhynchus mykiss*). Aquaculture.

[14] Shokri M., Francesco C., Fabio V., Marco B., Elisabetta P., Alberto B. (2022). Metabolic rate and climate change across latitudes: Evidence of mass-dependent responses in aquatic amphipods. J. Exp. Bio..

[15] Tsuda Y., Yamamoto S., Yamaguchi H., Ohnishi T., Sakamoto W., Murata O. (2014). Vertical movement of spawning cultured chub mackerel (*Scomber japonicus*) in a net-cage. Aquaculture.

[16] Jansen T., Post S., Olafsdottir A.H., Reynisson P., Óskarsson G.J., Arendt K.E. (2019). Diel vertical feeding behaviour of Atlantic mackerel (*Scomber scombrus*) in the Irminger current. Fish. Res..

[17] Varela J.L., Spares A.D., Stokesbury M.J.W. (2020). Feeding ecology of Atlantic bluefin tuna (*Thunnus thynnus*) in the Gulf of Saint Lawrence, Canada. Mar. Environ. Res..

[18] Takahashi M., Tamura T., Bando T., Konishi K. (2022). Feeding habits of Bryde’s and sei whales in the western North Pacific inferred from stomach contents and skin stable isotope ratios. J. Sea Res..

[19] Wiujamson C.E. (1984). Laboratory and field experiments on the feeding ecology of the cyclopoid copepod, *Mesocyclops edax*. Freshwater Biol..

[20] Cathcart K., Shin S.Y., Milton J., Ellerby D. (2017). Field swimming performance of bluegill sunfish, *Lepomis macrochirus*: Implications for field activity cost estimates and laboratory measures of swimming performance. Ecol. Evol..

[21] Huang M., Ding L., Wang J., Ding C., Tao J. (2021). The impacts of climate change on fish growth: A summary of conducted studies and current knowledge. Ecol. Indic..

[22] Egger D. (1977). Factors in interpretion data obtained by diel sampling of fish stomachs. Fish. Res. B. Can..

[23] Rezende E.L., Bozinovic F. (2019). Thermal performance across levels of biological organization. Biol. Sci..

[24] Shokri M., Ciotti M., Gjoni V., Basset A. (2019). Components of standard metabolic rate variability in three species of gammarids. Web Eco.

[25] Riche M., Haley D.I., Oetker M., Garbrecht S., Garling D.L. (2004). Effect of feeding frequency on gastric evacuation and the return of appetite in tilapia *Oreochromis niloticus* (L.). Aquaculture.

[26] Dürrani Ö., Seyhan K. (2019). Gastric evacuation rates in farmed brook trout subjected to a range of feeding conditions fed commercial pellets. Aquaculture.

[27] Moran C.J., Ferry L.A., Gibb A.C. (2016). Why does Gila elegans have a bony tail? A study of swimming morphology convergence. Zoology.

[28] Stavrakidis-Zachou O., Papandroulakis N., Lika K. (2019). A DEB model for European sea bass (*Dicentrarchus labrax*): Parameterisation and application in aquaculture. J. Sea Res..

[29] Sadoul B., Geffroy B., Lallement S., Kearney M. (2020). Multiple working hypotheses for hyperallometric reproduction in fishes under metabolic theory. Ecol. Model..

[30] Sigourney D.B., Letcher B.H., Obedzinski M., Cunjak R.A. (2008). Size-independent growth in fishes: Patterns, models and metrics. J. Fish Biol..

[31] Denderen D., Gislason H., Heuvel J., Andersen K.H., Leprieur F. (2020). Global analysis of fish growth rates shows weaker responses to temperature than metabolic predictions. Global Ecol. Biogeogr..

[32] Kooijman S.A.L.M., Andersen T., Kooi B.W. (2004). Dynamic Energy Budget Representations of Stoichiometric Constraints on Population Dynamics. Ecology.

[33] Go S., Lee K., Jung S. (2020). A Temperature-Dependent Growth Equation for Larval Chub Mackerel (*Scomber japonicus*). Ocean Sci. J..

[34] Tyler A. (1970). Rates of gastric emptying in young cod. Fish. Res. B. Can..

[35] Bernreuther M., Temming A., Herrmann J.P. (2009). Effect of temperature on the gastric evacuation in sprat *Sprattus sprattus*. J. Fish Biol..

[36] Beltramino L.E., Venerus L.A., Trobbiani G.A., Wilson R.P., Ciancio J.E. (2019). Activity budgets for the sedentary Argentine sea bass *Acanthistius patachonicus* inferred from accelerometer data loggers. Austral Ecol..

[37] Guillen A.C., Borges M.E., Herrerias T., Kandalski P.K., de Souza M., Donatti L. (2022). Gradual increase of temperature trigger metabolic and oxidative responses in plasma and body tissues in the Antarctic fish *Notothenia rossii*. Fish Physiol. Biochem..

[38] Brownscombe J.W., Raby G.D., Murchie K.J., Danylchuk A.J., Cooke S.J. (2022). An energetics-performance framework for wild fishes. J. Fish Biol..

